# Dysarthria and Altered Mental Status Following Electroconvulsive Therapy (ECT) in a Patient With Polycythemia Vera: A Case Report

**DOI:** 10.7759/cureus.73941

**Published:** 2024-11-18

**Authors:** Jordan Intrator, Jack Noto, Muhammad Abbas

**Affiliations:** 1 Neuropsychiatry, NYU Langone Health, New York, USA; 2 Psychiatry, Hackensack Meridian School of Medicine, Nutley, USA

**Keywords:** cerebrovascular accident (stroke), electroconvulsive therapy (ect), generalised anxiety disorder, geriatric psychiatry, hematology, neuromodulation, polycythemia vera, psychotic depression, rare cause of altered mental status

## Abstract

Although rare, neurological complications following electroconvulsive therapy (ECT) can have significant consequences. Recognizing patient populations that may be at high risk of neurological complications following ECT can reduce the likelihood of these patients developing such complications. We report a case of a patient with a history of polycythemia vera presenting with a worsening episode of depression with psychotic features who demonstrated a significant change in neurological status following ECT, suspected to be due to cerebral ischemia. This case report highlights the risk factors for cerebral ischemia in patients with polycythemia vera while undergoing ECT and provides important clues that may assist in distinguishing neuropsychiatric disturbances associated with polycythemia vera from the complications of a primary psychiatric illness.

## Introduction

Electroconvulsive therapy (ECT) is a highly effective therapy for depression, with remission rates of 55% or greater reported in some studies. Efficacy has been found to be even higher in patients with major depressive disorder with psychotic features, with one study demonstrating a 95% remission rate and remission occurring sooner [1]. ECT is also a safe intervention in all age groups that has no absolute contraindications and few relative contraindications [2,3]. The most common neurologic complications of ECT include transient memory loss and delirium, without evidence of long-term complications or memory impairment [4].

While rare, there are reports of cerebral ischemia development following ECT [5-7]. These cases have been attributed to particular risk factors, such as prior ischemic events, and acute changes in cerebral blood flow intra- and post-ictally in patients undergoing ECT [8,9]. Identifying patients with heightened risk factors for cerebral ischemia can improve screening for high-risk candidates that are under consideration to receive ECT.

Polycythemia vera is a hematological condition that increases the risk for cerebral ischemia [10,11]. It is a disease characterized by tumorigenic production of blood cells in the bone marrow [12]. The risk of ischemia is thought to be the consequence of blood hyperviscosity, the result of an increased density of blood cells impairing blood flow and cerebral oxygenation [13]. Ischemic events are often the first recognizable manifestation of the disease leading to its discovery [11].

Polycythemia vera can also present solely as a neuropsychiatric illness, which is thought to be the consequence of cerebral ischemia. In these rare cases, patients initially present with confusion, behavioral and psychomotor changes, and other signs of cognitive impairment [14,15]. These patients may be mistaken as having a primary mood disorder, especially when a corresponding ischemic event is not obvious. Neuropsychiatric symptoms are refractory to classic treatments like antidepressants, and often only relieved following phlebotomy and/or cytoreduction [14-16].

The neuropsychiatric symptoms of polycythemia vera can be difficult to distinguish from a primary psychiatric illness. Here, we present a case of a patient with a history of polycythemia vera and severe depression with psychotic features with worsening symptoms requiring psychiatric admission who exhibited significant neurologic deficits following ECT. This case demonstrates the need for awareness of the neuropsychiatric disturbances associated with polycythemia vera and provides insights into distinguishing these complications from those of a primary psychiatric illness.

## Case presentation

An 81-year-old Caucasian female patient with a past medical history of polycythemia vera and a past psychiatric history of anxiety and depression with psychotic features was admitted to the hospital for worsening auditory hallucinations in the context of a depressive episode over the course of five weeks. Polycythemia vera was diagnosed over two years prior and controlled with hydroxyurea 500 mg twice daily. Anxiety and depression are chronic issues, with auditory hallucinations emerging gradually at an unknown time in older age. Her hallucinations worsened suddenly without any precipitating factors. The patient denied suicidal ideation, homicidal ideation, visual hallucinations, or history of mania.

At the time of admission, the patient was being treated with sertraline 200 mg daily, alprazolam 0.75 mg every night, and risperidone 1 mg daily, which was added a week prior to admission. Her medical workup was unremarkable, and the patient was admitted to the psychiatric unit. Following admission, risperidone was increased to 2 mg daily, but was immediately reduced following the emergence of parkinsonian symptoms, with antipsychotic reduction to 1 mg and the addition of benztropine 20 mg daily. With symptoms continuing to persist and showing resistance to pharmacotherapy, the patient consented to undergo four ECT sessions every other day for one week. ECT treatments were right unilateral, with the EEG duration ranging from 50 to 70 seconds. Following the fourth ECT treatment, the patient initially showed no signs of significant side effects. However, overnight she developed dysarthria, disorientation, and agitation. Her blood oxygen saturation decreased to 89%.

A workup following the onset of these new symptoms consisted of chest X-ray, chest CT with angiography, CT of the head, MRI of the brain, MR angiography of the brain, urinalysis, complete metabolic panel, complete blood count, arterial blood gas test, electroencephalography, and testing for COVID-19. Results from the remainder of tests came back unremarkable and within the normal range. Labs were not concerning for an acute exacerbation of polycythemia vera, with the red blood cell count at 3 × 10^6^/uL, hemoglobin at 11.5 g/dL, and hematocrit 34.7%. The patient was treated with empiric antibiotics for possible occult infection for over 10 days, with minimal improvement in alertness and speech, but no improvement in confusion and orientation. Following this acute change in mental status, the patient could no longer provide consent and, at the request of the patient’s family, no further ECT treatments were administered. She was discharged 32 days after admission, 12 days following her last ECT treatment, to a subacute rehabilitation facility and was lost to follow-up.

## Discussion

Given the patient’s older age and worsening depression with psychotic features despite treatment with both antipsychotics and antidepressants, it is reasonable to ask whether her symptoms were of primary psychiatric origin or rather progression of complications of her hematologic condition. Prior reports suggest that neuropsychiatric presentations of polycythemia vera almost exclusively occur following the fifth decade of life [17]. Furthermore, it is reasonable to contextualize her treatment resistance in the context of her condition. Prior reports of neuropsychiatric presentations of polycythemia vera demonstrate its resistance to psychiatric pharmacotherapy [14,15,18]. If her neuropsychiatric disturbance was the result of polycythemia vera, then it is logical that her symptoms would not fully abate with therapies that do not address the underlying problem. Indeed, there are several case reports of neuropsychiatric symptom resolution when the hematological condition is treated, such as with therapeutic phlebotomy or cytoreduction [14-16].

Although our patient did not demonstrate abnormal hematological lab findings, cases of symptomatic polycythemia vera with routine blood count measurements have been reported in the literature [19]. Possible explanations for her non-elevated blood counts include masked polycythemia vera, the presence of concomitant anemia or anemia secondary to blood dilution [19-21]. Furthermore, previous reports demonstrate no correlation between neuropsychiatric symptoms and hematological parameters [17]. Although there is some evidence to suggest cytoreduction is superior to phlebotomy in patients with unremarkable hematological parameters [22], our patient developed neuropsychiatric symptoms whilst receiving cytoreduction. Therefore, despite what the labs may show, the literature would seem to support trialing venesection [14,23].

Decreasing the hematocrit level to below 45% is the primary indication for therapeutic phlebotomy in polycythemia vera [24]. Although our patient did not meet this need, cases of symptom amelioration and improved outcomes following phlebotomy in polycythemia vera with normal blood count findings have been reported in the literature [19,20]. Complications following venesection most often include hypovolemia and anemia [22]. However, given the patient's poor response to psychiatric treatment, and the development of treatment complications, phlebotomy is a relatively low-risk alternative.

Our patient’s latent neurological deficits following the fourth ECT treatment raises the question of whether her symptoms may have been the result of cerebral ischemia. While the differential reasonably includes many additional etiologies for these neurological deficits, including postictal delirium [25], aspiration pneumonia [26], metabolic encephalopathy [27], and tardive seizures [28], there are specific aspects of this case that raise particular concerns for inadequate cerebral perfusion.

Polycythemia vera has been known to cause hyperviscous blood flow leading to impaired cerebral perfusion [15]. ECT also affects cerebral blood flow, initially triggering a parasympathetic response followed by a sustained sympathetic response with an associated increase in the heart rate and blood pressure [5]. Although this results in an increase in cerebral blood flow for the duration of the procedure, it can cause a longer lasting decrease afterwards [8,29]. This, in the context of blood hyperviscosity, could have exacerbated already reduced cerebral perfusion [14,15,30]. Although the imaging findings in this patient were unrevealing, this result is consistent with previous reports of similar cases of neurological manifestations in polycythemia vera attributable to reduced cerebral oxygenation [14,15]. The persistence of our patient’s deficits up to 10 days following the onset favors this etiology. Although delirium following ECT is widely reported in the literature, it typically resolves within 5 to 45 minutes [31]. Prolonged delirium has been reported, however, to our knowledge, in only one case where post-ECT delirium lasted more than 48 hours [32], and this case was complicated by the presence of substance-induced psychosis, extensive pesticide exposure, and ischemic changes. Thus, we theorize that successive courses of ECT led to decreased cerebral blood flow, exacerbated by underlying blood hyperviscosity, resulting in stasis and cerebral ischemia.

## Conclusions

It is evident that neuropsychiatric symptoms of polycythemia vera can be disguised as a primary psychiatric illness, especially in the context of normal hematological parameters. Therefore, suspicion for neuropsychiatric polycythemia vera should remain high in any patient with the condition, especially in the older population and in those with treatment-resistant symptoms. Finally, caution should be taken when considering ECT in this patient population due to the risk of exacerbating cerebral ischemia. Venesection or cytoreduction should be considered among the first-line treatment procedures in this patient population as it addresses a likely underlying cause for neuropsychiatric disturbances. In patients with normal hematological findings, cytoreduction may be superior; however, venesection could be trialed for unresponsive patients. This case should create awareness about the complications that this population is at risk of and help reduce potential adverse events. Ultimately, however, further research is needed to better understand the risks associated with ECT in polycythemia vera.
